# The passive CNV: carving out the contribution of task-related processes to expectancy

**DOI:** 10.3389/fnhum.2013.00827

**Published:** 2013-12-12

**Authors:** Giovanni Mento

**Affiliations:** Department of General Psychology, University of PaduaPadova, Italy

**Keywords:** CNV, expectancy, motor preparation, S1–S2, passive oddball, decision-making

## Abstract

In this perspective article, I summarized certain theoretical and methodological issues concerning the investigation of the contribution of cognitive and motor processes to the electrophysiological stimulus-preceding activity. In particular, the question of whether the contingent negative variation (CNV) is a marker reflecting both cognitive expectancy and motor preparation in the S1–S2 paradigms was discussed. New evidence suggests that it is possible to isolate an automatic temporal expectancy-related cognitive mechanism relying on a passive CNV after ruling out the contribution of task-related processes, including motor and decisional processes, to it. This can be achieved by simply manipulating the trial temporal structure according to a probabilistic, oddball distribution. The scientific value of this finding is framed within a historical perspective in the attempt to bridge together the classic literature linking the CNV to stimulus preparation and the more recently published literature linking the CNV to temporal processing.

## The discovery of the contingent negative variation (CNV): a milestone for modern psychophysiology

In 1964, Grey Walter and his colleagues published their pioneering work on a psychophysiological phenomenon that is nowadays commonly known as the contingent negative variation (CNV; Walter et al., 1964). This study had the following two main values: one historical and one scientific. The first one is extremely important in the field of psychophysiology because it marked the advent of a modern era of research on cognitive event-related potentials (ERPs). In fact, at that time, research on ERP was limited to the sensory stimulus-locked activity, neglecting the existence of a large amount of phenomena dealing with stimulus-preceding activity. The second one described, for the first time, a specific and basic phenomenon framed in between the field of neurophysiology and the field of “Cognitive Neuroscience”. This involves the ability of the central nervous system of primates and other species to produce anticipatory activity against important events. In fact, everyday phenomenological evidence suggests that many species possess the ability to anticipate and prepare for events in the environment on the basis of special cues that are both internal (endogenous) and/or in the environmental structure itself (exogenous). However, until the study by Walter et al. (1964) the physiological basis that governs this mechanism remained unknown. Walter et al. (1964) presented participants with couples of stimuli, commonly referred as S1–S2, which consisted of a warning signal followed by a target stimulus after a variable foreperiod in the range of few seconds. When subjects were instructed to press a button to detect the target, a large negative voltage arose at frontocentral electrode sites soon after the offset of S1 and peaked just before the onset of S2. As it was immediately clear that this negative ERP component did not reflect any sensory activity, the authors argued that it could be directly associated with something having a more abstract nature, which dealt with the ability to represent the causal, contingent link between two stimuli.

## What exactly does the CNV reflect?

 A bulk of work describing the characteristics of CNV in relation to specific variables such as probability, intensity, interstimulus interval (ISI), motivation, arousal, intentionality, etc., has been published in the previous decades (Irwin et al., 1966; Cant and Bickford, 1967; Tecce, 1972; Loveless and Sanford, 1975; Näätänen et al., 1977; Birbaumer et al., 1990; Brunia, 1993; Brunia and van Boxtel, 2001). One of the most debated issues concerns the exact process that is reflected by the CNV. Initial claims interpreted this electrophysiological index as being inextricably related to both cognitive- and motor-related processes. In fact, the preparation for action may induce a concurrent expectation of when that action is likely to be executed (Frost et al., 1988; Requin et al., 1991). At the same time, expectation of an event’s onset may automatically instantiate a temporal tuning for preparing the motor effector suitable for responding to that event (Gibson, 1979; Cotti et al., 2011). The link between the representational processes related to expectancy and those that are more strictly action-directed was indeed strongly suggested by the title of the Walter et al. (1964) original report. In this well-known study, Walter et al. (1964) manipulated several experimental variables, including the stimuli (i.e., clicks or flashes), the contingency between S1 and S2 (i.e., the probability of the association between the conditional and the imperative stimulus), and the mental attitude of subjects who respond to S2 (i.e., the intentionality of the response). The first result they reported was that a reliable CNV was present only when S2 required a button press. In fact, when S1 and S2 were presented alone or in couple but without any clear S2-related imperative response, the CNV was not elicited. The explanation of this phenomenon relies on the fact that the CNV is generated only when a contingency relationship between stimulus and response is established. A second interesting finding was that once a S1–S2 contingency was established, a clear CNV could be elicited in the absence of a response, that is, when S2 was not presented at all. Nevertheless, in this case, a progressive extinction of the CNV occurred after approximately 30 trials. As outlined by the authors, this data accounted for the CNV being dependent on the subjective probability of S2 occurrence at a given moment. Thus, if S2 is repeatedly omitted, the subjective probability of the onset of S2 is disrupted, in turn, causing a progressive CNV extinction. As a third remarkable finding, Walter et al. (1964) showed that when subjects were asked to deliberately decide on a trial-by-trial basis to respond to S2 or not, the CNV was reliably elicited only when they in fact pressed the button. Remarkably, this occurred despite the fact that probabilistic contingency and the motor response were maintained. This last finding led them to hypothesize that the CNV could also strongly depend on the intentionality of the action. Finally, Walter et al. (1964) showed that a CNV was exhibited even when subjects were asked to perform a pure mental judgment of a time interval in the total absence of an operant response. This would suggest that the CNV may also reflect decisional processes in addition to preparatory processes. Based on these findings of Walter et al. (1964), the CNV reflects a multicomponential process in which the interplay among motor, intentional, and decisional task-related components underlying stimulus-preceding activity cannot be easily disentangled.

The attempt to better characterize the CNV was a topic of research in the decades following the study of Walter et al. (1964). Much work has been done in the 70’s and 80’s to determine the role of different cognitive and motor components in subserving the CNV generation (for a review, see Tecce and Cattanach, 1993). By increasing the S1–S2 to more than 3 s, Weerts and Lang (1973) were able to demonstrate that the CNV was composed of two main components. The first wave arose early after the offset of the warning stimulus and was called the orienting wave or the “O” -wave. Instead, the second wave appeared to be the most prominent immediately before the imperative stimulus and was called the expectancy wave or the “E” -wave (Rohrbaugh and Gaillard, 1983). Besides showing a different temporal pattern, these two components displayed a different scalp location, as the O-wave was more frontally distributed, whereas the E-wave was precentrally located (Gaillard, 1976). Other evidence has accumulated suggesting that the O- and E-waves may reflect distinct and dissociable processes. For example, the E-wave is more sensitive to the anticipation of an event that has yet to occur, whereas the O-wave may occur in response to a previously presented item. This achievement ultimately suggested that the early portion of the CNV may be more related to a “pure” cognitive process not necessarily related to the action performed. However, the late terminal E-wave component of the CNV appeared to be determined mainly by the level of motor preparation (Rohrbaugh et al., 1986).

Following the original suggestion of Walter et al. (1964), later studies attempted to elicit a CNV in the absence of a motor response to temporally disentangle between the E- and O-waves. This was undertaken by omitting S2 (Jarvilehto and Fruhstorfer, 1970; Nakamura et al., 1975; Ruchkin et al., 1986) or by cueing subjects not to respond to it. For instance, one study originally designed to investigate the effect of ISI probability distribution on CNV showed that in NoGo trials, this last peaked at the end of the most probable ISI according to an *a posteriori* rather than *a priori* probability (Trillenberg et al., 2000). However, although the elicitation of the CNV without a motor response constitutes a consolidated data, it should be taken into account that this finding is usually obtained when participants are somehow instructed to not respond in only some experimental conditions (e.g., according to a NoGo cue) after establishing a contingency rule (see above). This ultimately implies that even the suppression of CNV may still depend on the decision that a given action should not be performed. As a logical implication, although the contribution of execution and/or preparatory motor components can be eliminated in particular cases, it is still difficult to determine whether and to what extent the CNV may mirror additional task-related processes, including response selection, decision making, and intentionality.

## The temporal CNV

In addition of being related to stimulus anticipatory activity, the CNV has also consistently been shown to be a reliable electrophysiological hallmark of timing. In particular, the CNV has been reported in temporal production (Macar et al., 1999; Macar and Vidal, 2003) or reproduction (Elbert et al., 1991; Kononowicz and van Rijn, 2011) tasks. It has also been claimed that distinct neural generators underlie the CNV in relation to the implicit or explicit nature of the temporal task. In explicit timing tasks, the supplementary motor area (SMA) would be the most probable candidate as the CNV generator (Macar et al., 1999). In contrast, in implicit timing tasks, the CNV appeared to predominantly originate from the bilateral premotor cortex (Praamstra et al., 2006). One main explanation linking the temporal CNV with activation in the premotor area is the fact that event predictability may trigger anticipatory, action-directed neural activity resulting in optimization of behavior, such as faster reaction times. As a matter of fact, the evidence that multiple neural generators underlie the CNV makes it even more difficult to determine the contribution of a pure representational expectancy component to that action-related or, at least, to temporally disentangle them.

A clear CNV has also been observed even when motor requirements are minimized, for example, as that observed in temporal discrimination tasks (Macar et al., 1999; Pfeuty et al., 2005; Tarantino et al., 2010) based on the “two-alternative forced choice method” (Grondin and Rousseau, 1991). In this case, instead of producing or re-producing a given duration, participants are asked to judge whether a probe interval is longer, equal, or shorter than a previously stored target interval. The interesting finding is that the CNV morphological features (i.e., peak and slope inversion latency) mirror the duration of a previously encoded target interval when it has to be compared with an ongoing duration. Given that in this case, the motor response is usually delayed after a comparison has been made, the CNV can be interpreted here as reflecting a perceptual rather than a motor temporal discrimination ability.

The evidence that the CNV can be generated across a wide range of motor and perceptual temporal tasks led to the assumption that it is a general neural signature of time processing (Macar and Vidal, 2009). This hypothesis would support the most influential theoretical account of interval timing, that is, the pacemaker–accumulator (PA) model (e.g., Treisman, 1963; Gibbon, 1977; Gibbon et al., 1984). This three-step model postulates the existence of an “internal clock” marking out time through the combined activity of a pacemaker that emits pulses and an accumulator that collates and integrates such pulses (first step). These pulses are then stored in the working memory and compared with a previously encoded duration (second step) to make a decision (third step). Given the similarity between the morphological features of CNV and the hypothesized characteristics of the accumulation process, the CNV would reflect the activity of the neural substrate of the temporal accumulator (Macar and Vidal, 2004). According to this assumption, the CNV amplitude and slope would predict the behavioral performance at a single-subject level. Furthermore, in line with the PA model, one should not expect habituation effects on CNV morphology. However, both these assumptions have been recently challenged by a study that failed to replicate the expected results according to the PA model (Kononowicz and van Rijn, 2011). Alternative hypotheses have been recently put forward that interpreted the CNV as an index of temporal preparation (Ng et al., 2011) and/or decision (Kononowicz and van Rijn, 2011) rather than accumulation (for a recent discussion, see Van Rijn et al., 2011). However, one important consideration to be noted is that in temporal tasks, both preparation and decision are intrinsically “temporally-driven”. That is, whatever the task performed to elicit the temporal CNV, the act of preparing necessarily implies that “something” has to be undertaken over time, such as pressing a button at the right time. Similarly, the act of discriminating between two temporal durations necessarily implies the use of time to make a decision. Hence, whether and to what extent the CNV can underlie a “core” temporal mechanism related to expectancy regardless the contribution of other task-related processes, such as time-based preparation and decision, remains as an incompletely answered question.

## The passive CNV

**Figure 1:**
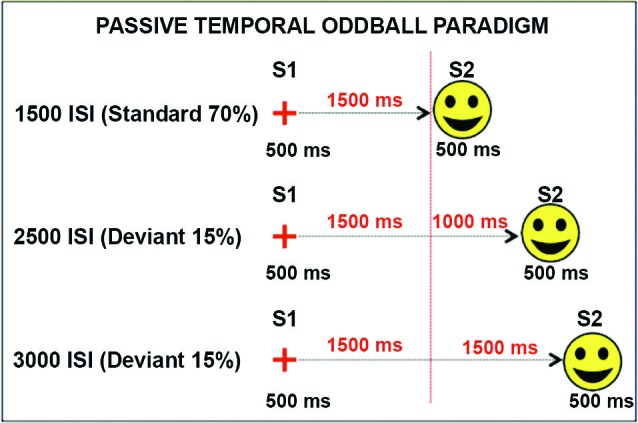
**Passive temporal oddball task**. S1 and S2 never changed across conditions. The only manipulated variable was the ISI delay. In 70% of trials, the ISI was 1500 ms (1500 standard condition). In the remaining 30% of trials, the ISI was lengthened to 2500 or 3000 ms (2500 and 3000 deviant conditions, 15% each). Standard and deviant conditions were randomly presented. Participants were given neither instruction nor a motor task. The vertical dotted red line represents the S2 maximum expectation time point corresponding to the end of the standard ISI.

**Figure 2:**
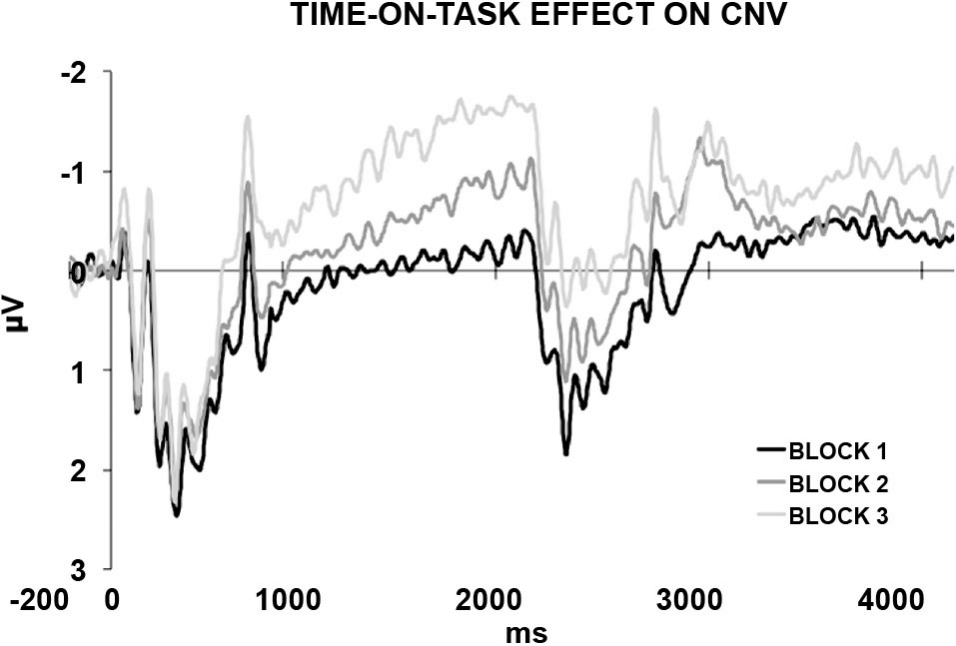
**The passive CNV.** The generation of a temporal S1–S2 contingency rule instantiates an automatic temporal expectancy. This in turn results in the elicitation of a passive CNV peaking at the expected S2 time point, corresponding to approximately 2000 ms from the onset of the stimulus, that is, S1 (500 ms) + standard ISI (1500 ms). The standard ISI-related ERP activity is plotted separately for the first, second, and third block. The progressive increases in amplitude and steepness reveals a time-on-task effect. This suggests that participants learnt the temporal structure progressively, discovering the temporal contingency between S1 and S2 on a trial-by-trial basis.

In a recently published study (Mento et al., 2013), we advanced the idea that the optimal manner to disentangle between the contribution of task-related processes and temporal expectancy in building up the CNV would have been to completely eliminate any S2-related processing. For this purpose, a new hybrid, passive task was designed by merging two well-known experimental paradigms, that is, the S1–S2 paradigm usually used to elicit a CNV and the classic oddball paradigm used to elicit deviant-related ERP responses (Figure 1). This task, called “passive temporal oddball task”, enables the progressive generation of a strong automatic temporal expectancy of S2, although no actions are in fact associated with it. The hypothesis was that by simply exploiting an unbalanced statistical distribution of the ISI, the subjects could become passively and progressively attuned to a standard S1–S2 interval. An oddball probabilistic distribution (e.g., 70% for the standard and 30% for the deviant ISIs) was therefore used to implicitly generate a temporal contingency between S1 and S2. In fact, the idea to use a passive oddball paradigm to investigate the effect of ISI manipulation was not new. However, literature conventionally focussed on the stimulus-locked ERP activity, including the Mismatch Negativity (MMN; Brannon et al., 2004), rather than the slow ERP activity during the ISI itself. The rationale is that if the “expectancy” component of the CNV can be dissociated from any action-related mechanism, then we should expect this component to be elicited even in the absence of any task. By contrast, if the CNV necessarily brings itself some sort of action- or decision-related mechanism (either related to preparing, executing, withholding, or selecting a response), we should expect not to find any specific stimulus-preceding activity in the absence of a clear task associated with S2. Consistent with the first assumption, the results confirmed the presence of a specific ISI-related ERP activity consisting of a slow negative, centrally located deflection, called “passive CNV”, initiating after the end of the S1 and peaking at the end of the standard ISI when S2 expectancy was maximum. Even more interestingly, the passive CNV was shown to be sensitive to time-on-task effects, becoming steeper block-by-block (Figure 2). This data support the idea that participants discovered the temporal structure progressively, learning the temporal link between S1 and S2 on a trial-by-trial basis.

The elicitation of a passive CNV may be believed to account for a specific, elementary cognitive mechanism. This relies on the fact that when we are allowed to infer an environmental temporal regularity establishing an implicit association between contingent events, an automatic expectancy is progressively generated even when this operation is not aimed at performing any specific task. This mechanism probably deals with the innate capacity of our cognitive system to track event regularities over time to build up an internal representational model (i.e., a predictive code) of the external environment and to update it according to externally or internally generated rules with the final aim of predicting events (Friston, 2010; but see Clark, 2013 for an exhaustive discussion). Given that this mechanism appears to occur even when no specific action or task is required, it may be speculated that it is action-independent in nature, although further evidence is needed to support this view.

## Conclusion

The CNV is one of the most well-known psychophysiological phenomenon. It has been proposed that the CNV depends on the fact that cognitive and motor processes usually intertwine when anticipating the occurrence of a stimulus, although it is not entirely understood to what extent they differentially concur to its generation. The main source of confusion arises from the fact that CNV has been usually investigated with paradigms engaging in different motor and cognitive demands. Nevertheless, manipulating trial structure enables the isolation of a core, expectancy-related cognitive mechanism. This relies on the elicitation of a passive CNV. This finding represents a first step toward a deeper understanding of the subtle relationship between representational and motor contribution on event preparation.

## Conflict of interest statement

The author declares that the research was conducted in the absence of any commercial or financial relationships that could be construed as a potential conflict of interest.
